# Prognostic value of coronary vascular dysfunction assessed by rubidium-82 PET/CT imaging in patients with resistant hypertension without overt coronary artery disease

**DOI:** 10.1007/s00259-021-05239-w

**Published:** 2021-02-16

**Authors:** Valeria Gaudieri, Teresa Mannarino, Emilia Zampella, Roberta Assante, Adriana D’Antonio, Carmela Nappi, Valeria Cantoni, Roberta Green, Mario Petretta, Parthiban Arumugam, Alberto Cuocolo, Wanda Acampa

**Affiliations:** 1 Department of Advanced Biomedical Sciences, University Federico II, Via Pansini 5, 80131 Naples, Italy; 2 IRCCS SDN, Naples, Italy; 3 Department of Nuclear Medicine, Central Manchester Foundation Trust, Manchester, UK; 4 Institute of Biostructure and Bioimaging, National Council of Research, Naples, Italy

**Keywords:** PET/CT, Myocardial perfusion reserve, Resistant hypertension, Prognosis

## Abstract

**Purpose:**

The identification of coronary vascular dysfunction may enhance risk stratification in patients with resistant hypertension (RH). We evaluated if impaired coronary vascular function, assessed by rubidium-82 (^82^Rb) positron emission tomography/computed tomography (PET/CT) imaging, is associated with increased cardiovascular risk in patients with hypertension without overt coronary artery disease (CAD).

**Methods:**

We studied 517 hypertensive subjects, 26% with RH, without overt CAD, and with normal stress-rest myocardial perfusion imaging at ^82^Rb PET/CT. The outcome end points were cardiac death, nonfatal myocardial infarction, coronary revascularization, and admission for heart failure.

**Results:**

Over a median of 38 months (interquartile range 26 to 50), 21 cardiac events (4.1% cumulative event rate) occurred. Patients with RH were older (*p* < 0.05) and had a higher prevalence of left ventricular hypertrophy (*p* < 0.001), a lower hyperemic myocardial blood flow (MBF), and myocardial perfusion reserve (MPR) (both *p* < 0.001) compared to those without. Conversely, coronary artery calcium content and baseline MBF were not different between patients with and without RH. At univariable Cox regression analysis, age, RH, left ventricular ejection fraction, coronary artery calcium score, and reduced MPR were significant predictors of events. At multivariable analysis, age, RH, and reduced MPR (all *p* < 0.05) were independent predictors of events. Patients with RH and reduced MPR had the highest risk of events and the major risk acceleration over time.

**Conclusion:**

The findings suggest that the assessment of coronary vascular function may enhance risk stratification in patients with hypertension.

## Introduction

Hypertension is the leading risk factor for stroke, cardiovascular disease, and premature death [1]. Patients with treatment-resistant hypertension (RH) have a higher risk of hypertension-mediated organ damage and a Framingham 10-year coronary risk score > 20% [1]. Moreover, RH patients present more severe alterations of vascular function compared to patients with controlled hypertension (CH), as supported by the high rates of peripheral atherosclerosis, reduced endothelial function, impaired arterial compliance, and elevated systemic vascular resistance [2]. Rubidium-82 (^82^Rb) positron emission tomography (PET)/computed tomography (CT) allows the quantification of myocardial blood flow (MBF) at rest and during vasodilator stress, providing a non-invasive evaluation of myocardial perfusion reserve (MPR) [3, 4]. By integrating epicardial and microvascular circulations, MPR depicts the vasodilator capacity of the coronary circulation. The added value of MBF and MPR has been demonstrated in the identification of impaired coronary vascular function and also as an indicator of therapeutic interventions effectiveness [5] and in the prognostic assessment of the patient with suspected and known coronary artery disease (CAD) [6–8]. Previous studies demonstrated that patients with RH compared with patients with CH have a lower value of coronary flow reserve assessed by transthoracic Doppler echocardiography [9] and lower hyperemic MBF and MPR evaluated by ^82^Rb PET/CT [10]. However, in patients with RH, the prognostic value of the coronary vascular function in predicting cardiovascular events has been never investigated. This study was designed to evaluate whether measurement of coronary vascular function by ^82^Rb PET/CT helps in predicting outcome in patients with hypertension and without overt CAD.

## Materials and methods

### Patients

From March 2012 and March 2014, 2075 hypertensive patients underwent stress-rest ^82^Rb PET/CT as part of their diagnostic workup. For the purpose of the present investigation, 1237 hypertensive patients with known CAD, 166 with abnormal myocardial perfusion imaging, and 120 with heart failure were excluded. Follow-up data were not available in 35 (6%) of the remaining 552 patients, leaving 517 subjects for the analysis. For each patient, the presence of coronary risk factors was noted. Arterial hypertension was defined as repeated blood pressure (BP) measurements of ≥ 140 mmHg systolic and/or ≥ 90 mmHg diastolic and/or intake of antihypertensive medications [1]. Resistant hypertension (RH) was defined as hypertension that remains uncontrolled with three antihypertensive (including one diuretic) drugs, administered at maximum or maximally tolerated daily doses, or BP controlled on four medications [2]. Diabetes was defined when the patients had any one of the criteria as follows: fasting blood glucose ≥ 126 mg/dL, random blood glucose ≥ 200 mg/dL, blood glucose ≥ 200 mg/dL 2 h after a 75 g oral glucose tolerance test within the past 3 months, currently taking drugs to treat hyperglycemia, or prior medical diagnosis of diabetes. Hypercholesterolemia was defined as a total cholesterol level > 6.2 mmol/L or treatment with cholesterol-lowering medication. A positive family history of CAD was defined by the presence of disease in first-degree relatives younger than 55 years in men or 65 years in women. Patients were defined as symptomatic if they reported atypical angina and/or shortness of breath. The review committee of our institution approved this study and all patients gave informed consent (“Comitato Etico, Università Federico II”, protocol number 110/17).

### PET/CT imaging

As a routine preparation for ^82^Rb cardiac PET/CT, patients were asked to discontinue taking methylxanthine containing foods or beverages for 24 hours. In all patients, anti-hypertensive medications were not discontinued before imaging studies. Scans were acquired using Biograph mCT 64-slice scanners (Siemens Healthcare). Rest and stress cardiac PET/CT images were acquired as follows: scout CT was performed to check patient position and low-dose CT (0.4 mSv; 120 kVp; effective tube current, 26 mA [11-mAs quality reference]; 3.3 s) was performed for attenuation correction, during normal breathing before and after PET acquisitions. For both rest and stress imaging, 1110 MBq of ^82^Rb was injected intravenously with a 7-min list-mode PET acquisition. Dynamic PET acquisition was started at rest followed by an adenosine pharmacologic stress test (140 μg × kg^−1^ × min^−1^ for 4.5 min, with tracer injection between 2 and 2.5 min). Both rest and stress dynamic images were reconstructed into 26-time frames (12 × 5 s, 6 × 10 s, 4 × 20 s, and 4 × 40 s; total, 6 min) using the vendor standard ordered-subsets expectation maximization 3D reconstruction (2 iterations, 24 subsets) with 6.5-mm Gaussian post-processing filter. In addition, the images were corrected for attenuation using the low-dose CT. The heart rate, systemic BP, and 12-lead ECG were recorded at baseline and throughout the infusion of adenosine. External cardiac work was estimated as a rate-pressure product and was calculated as heart rate × systolic arterial BP. Myocardial perfusion, LV volumes, and EF were calculated using an automated software (QPS and QGS, Cedars-Sinai Medical Center, Los Angeles, CA, USA). LVEF reserve was computed as stress EF-rest EF [11]. Regional myocardial perfusion was evaluated, using standardized segmentation of 17 myocardial regions [12]. Each myocardial segment was scored from normal (score = 0) to absent perfusion (score = 4). The summed stress score was obtained by adding the scores of the 17 segments of the stress images. A similar procedure was applied to the resting images to calculate the summed rest score. The summed difference score was defined as the difference between the stress and rest scores. Myocardial perfusion finding was considered normal when summed stress score was < 3 and/or LVEF ≥ 45%.

Absolute MBF (in mL × min^−1^×g^−1^) was computed from the dynamic rest and stress imaging series with commercially available software (Siemens Syngo Dynamic PET) [13]. MPR was defined as the ratio of hyperemic to baseline MBF and was considered reduced when < 2 [14]. The MPR values were calculated using baseline MBF corrected for a rate-pressure product.

Coronary calcification was defined as a plaque with an area of 1.03 mm^2^ and a density ≥ 130 HU. Coronary artery calcium (CAC) score was calculated according to the method described by Agatston et al. [15]. Experienced nuclear medicine physicians analyzed the CT studies, blinded to the PET results. CAC score was calculated separately for the left anterior descending, left circumflex, and right coronary arteries, and summed to provide a total CAC score. CAC score was also categorized into 4 groups (0; 0.1–99.9; 100–399; and ≥ 400).

### Outcome

The follow-up was obtained by the use of a questionnaire that was assessed by a phone call to all patients and by the review of hospital or physicians’ records by individuals blinded to the patient’s test results. The outcome was a composite endpoint of cardiac death, nonfatal myocardial infarction, coronary revascularization, or admission for heart failure whichever occurred first. The cause of death was confirmed by a review of the death certificate, hospital chart, or physician’s records. Death was considered to be of cardiac origin if the primary cause was defined as acute myocardial infarction, congestive heart failure, valvular heart disease, sudden cardiac death, and cardiac interventional/surgical procedure related. Myocardial infarction was defined when > 2 of the following 3 criteria were met: chest pain or equivalent symptom complex, positive cardiac biomarkers, or typical electrocardiographic changes [16]. All hospitalization events occurred more than 30 days following imaging. The date of the last examination or consultation was used to determine the length of follow-up.

### Statistical analysis

Continuous data are expressed as mean ± standard deviation and categorical data as a percentage. A student two-sample *t* test and *χ*^2^ test were used to compare the differences in continuous and categorical variables, respectively. A *p* value < 0.05 (two-sided) was considered statistically significant. Hazard ratios (HR) with 95% confidence intervals (CI) were calculated by univariable and multivariable Cox regression analysis. Variables showing a *p* value < 0.05 at univariable analysis were considered for multivariable analysis. Event-free survival curves were obtained by the Kaplan-Meier method and compared using the log-rank test. Annualized event rates (AER) were calculated as the cumulative number of events divided by person-time and expressed as events per 100 person-years. A parametric survival model was used to identify which variables influenced time to event and to estimate risk-adjusted event rates during follow-up [17, 18]. Based on the distribution of survival times in our cohort, a Weibull distribution was selected for parametric survival, and a good fit was found. In this distribution, if the shape parameter > 1, the hazard rate increases with time; if < 1, the hazard rate decreases with time; and if = 1, the hazard rate is constant. All the analyses were performed using STATA version 14.0 for Windows (StataCorp LP, College Station, TX) and JMP (SAS Institute, Cary, North Carolina).

## Results

### Patient characteristics and imaging findings

Of the 517 patients enrolled, 136 (26%) had RH and 381 (74%) CH. Baseline patient characteristics and imaging findings according to hypertensive status are shown in Table 1. Patients with RH were slightly older and, as expected, had a higher prevalence of LV hypertrophy. Compared to patients with CH, those with RH had lower values of heart rate at peak of the stress test and higher values of systolic and diastolic BP both at rest and at peak of the stress test. A significant response to the stress test was observed in both hypertensive groups with a significant decrease in diastolic and systolic BP and a significant increase in heart rate. Yet, patients with RH had lower values of hyperemic MBF and MPR.

**Table 1:** Patient characteristics, medical treatment, hemodynamic data, and imaging findings according to hypertensive status

	All patients (*n* = 517)	RH (*n* = 136)	CH (*n* = 381)	*p* value
Age (years)	61 (12)	63 (13)	60 (12)	< 0.05
Male gender, *n* (%)	250 (48)	75 (55)	175 (46)	0.07
BMI (kg/m^2^)	31 (6)	30 (6)	31 (7)	0.25
Diabetes, *n* (%)	140 (27)	31 (23)	109 (28)	0.19
Hypercholesterolemia, *n* (%)	374 (72)	107 (79)	267 (70)	0.06
Smoking history, *n* (%)	155 (30)	30 (22)	125 (33)	< 0.05
Family history of CAD, *n* (%)	248 (48)	58 (43)	190 (50)	0.15
Symptoms, *n* (%)	313 (60)	70 (52)	243 (64)	< 0.05
LV hypertrophy, *n* (%)	207 (40)	77 (57)	193 (30)	< 0.001
Beta-blockers, *n* (%)	253 (49)	95 (70)	158 (42)	< 0.001
Calcium channel blockers, *n* (%)	222 (43)	113 (83)	109 (29)	< 0.001
Renin-angiotensin blockers, *n* (%)	330 (64)	126 (93)	204 (54)	< 0.001
Diuretics, *n* (%)	200 (39)	131 (96)	69 (18)	< 0.001
Rest HR (bpm)	70 (13)	69 (13)	71 (13)	0.25
Peak stress HR (bpm)	83 (16)*	81 (15)*	84 (16)*	< 0.05
Rest SBP (mmHg)	145 (20)	156 (22)	141 (19)	< 0.001
Peak stress SBP (mmHg)	133 (19)*	142 (20)*	130 (18)*	< 0.001
Rest DBP (mmHg)	85 (13)	91 (13)	83 (12)	< 0.001
Peak stress DBP (mmHg)	78 (12)*	82 (13)*	76 (11)*	< 0.001
Rest EDV (mL)	98 (34)	106 (34)	95 (34)	< 0.05
Peak stress EDV (mL)	107 (35)	114 (36)	105 (35)	< 0.05
Rest ESV (mL)	47 (23)	51 (24)	45 (23)	< 0.05
Peak stress ESV (mL)	48 (24)	53 (24)	47 (24)	0.06
Rest LVEF (%)	55 (8)	56 (8)	55 (8)	0.75
Peak stress LVEF (%)	57 (8)	56 (8)	57 (9)	0.54
LVEF reserve (%)	1.86 (3.68)	1.64 (3.82)	1.93 (3.63)	0.49
Baseline MBF (mL/min/g)	1.03 (0.27)	1.02 (0.26)	1.03 (0.27)	0.74
Hyperemic MBF (mL/min/g)	2.57 (0.82)*	2.18 (0.81)*	2.71 (0.77)*	< 0.001
MPR	2.56 (0.71)	2.16 (0.65)	2.71 (0.68)	< 0.001
MPR < 2, *n* (%)	111 (21)	63 (46)	48 (13)	< 0.001
CAC score categories				0.08
0, *n* (%)	280 (54)	61 (45)	219 (57)	
0.1–99.9, *n* (%)	73 (14)	24 (17)	49 (13)	
100–399.9, *n* (%)	65 (13)	19 (14)	46 (12)	
≥ 400, *n* (%)	99 (19)	32 (24)	67 (18)	

Values are expressed as mean value ± standard deviation or as number (percentage)

*RH* resistant hypertension, *CH* controlled hypertension, *BMI* body mass index, *CAD* coronary artery disease, *LV* left ventricular, *HR* heart rate, *SBP* systolic blood pressure, *DBP* diastolic blood pressure, *EDV* end-diastolic volume, *ESV* end-systolic volume, *EF* ejection fraction, *MBF* myocardial blood flow, *MPR* myocardial perfusion reserve, *CAC* coronary artery calcium

*p < 0.001 vs. baseline

### Outcome and predictors of events

Over a median follow-up of 38 months (interquartile range 26 to 50 months), 21 cardiac events (4.1% cumulative event rate) occurred: 3 cardiac deaths (14%), 4 myocardial infarctions (19%), 4 revascularization procedures (19%), and 10 admissions for heart failure (48%). Patients’ characteristics and imaging findings according to the occurrence of events are reported in Table 2. Patients with events, compared to those without, were older and had a higher prevalence of RH. A significant lower heart rate response to pharmacological stress test and LVEF reserve were observed in patients experiencing events. Patients with events also had a lower prevalence of CAC score 0 and lower values of hyperemic MBF and MPR.

**Table 2:** Patient characteristics, medical treatment, hemodynamic data, and imaging findings according to cardiac events

	All patients (*n* = 517)	Cardiac events (*n* = 21)	No cardiac events (*n* = 496)	*p* value
Age (years)	61 (12)	70 (14)	61 (12)	< 0.001
Male gender, *n* (%)	250 (48)	9 (43)	241 (49)	0.61
BMI (kg/m^2^)	31 (6)	32 (6)	31 (6)	0.4
Diabetes, *n* (%)	140 (27)	8 (38)	132 (27)	0.25
Hypercholesterolemia, *n* (%)	374 (72)	16 (76)	358 (72)	0.69
Smoking history, *n* (%)	155 (30)	7 (33)	148 (30)	0.73
Family history of CAD, *n* (%)	248 (48)	10 (48)	238 (48)	0.97
Symptoms, *n* (%)	313 (60)	14 (67)	299 (64)	0.56
Resistant hypertension, *n* (%)	136 (26)	13 (62)	123(25)	< 0.001
LV hypertrophy, *n* (%)	207 (40)	12 (57)	193 (39)	0.1
Beta-blockers, *n* (%)	253 (49)	15 (71)	238 (48)	< 0.05
Calcium channel blockers, *n* (%)	222 (43)	9 (43)	213 (43)	0.99
Renin-angiotensin blockers, *n* (%)	330 (64)	15 (71)	315 (63)	0.46
Diuretics, *n* (%)	200 (39)	15 (71)	185 (25)	< 0.005
Rest HR (bpm)	70 (13)	68 (9)	70 (14)	0.69
Peak stress HR (bpm)	83 (16)*	77 (10)*	84 (16)*	< 0.05
Rest SBP (mmHg)	145 (20)	151 (27)	144 (20)	0.14
Peak stress SBP (mmHg)	133 (19)*	136 (26)*	133 (19)*	0.47
Rest DBP (mmHg)	85 (13)	85 (18)	86 (13)	0.88
Peak stress DBP (mmHg)	78 (12)*	74 (16)*	78 (12)*	0.13
Rest EDV (mL)	98 (34)	104 (38)	98 (34)	0.43
Peak stress EDV (mL)	107 (35)	113 (38)	107 (35)	0.43
Rest ESV (mL)	47 (23)	51 (27)	47 (23)	0.45
Peak stress ESV (mL)	48 (24)	54 (26)	48 (24)	0.26
Rest LVEF (%)	55 (8)	54 (9)	55 (8)	0.42
Peak stress LVEF (%)	57 (8)	54 (9)	57 (8)	0.15
LVEF reserve (%)	1.86 (3.68)	0.2 (3.27)	1.95 (3.68)	< 0.05
Baseline MBF (mL/min/g)	1.03 (0.27)	1.06 (0.29)	1.03 (0.27)	0.67
Hyperemic MBF (mL/min/g)	2.57 (0.82)*	2.2 (0.71)*	2.59 (0.82)*	< 0.05
MPR	2.56 (0.71)	2.08 (0.66)	2.58 (0.7)	< 0.005
MPR < 2, *n* (%)	111 (21)	10 (48)	101 (20)	< 0.005
CAC score categories				< 0.05
0, *n* (%)	280 (54)	5 (24)	275 (56)	
0.1–99.9, *n* (%)	73 (14)	4 (19)	69 (14)	
100–399.9, *n* (%)	65 (13)	4 (19)	61 (12)	
≥ 400, *n* (%)	99 (19)	8 (38)	91 (18)	

Values are expressed as mean value ± standard deviation or as number (percentage)

*BMI* body mass index, *CAD* coronary artery disease, *LV* left ventricular, *HR* heart rate, *SBP* systolic blood pressure, *DBP* diastolic blood pressure, *EDV* end-diastolic volume, *ESV* end-systolic volume, *EF* ejection fraction, *MBF* myocardial blood flow, *MPR* myocardial perfusion reserve, *CAC* coronary artery calcium

*p < 0.001 vs. baseline

**Fig. 1:**
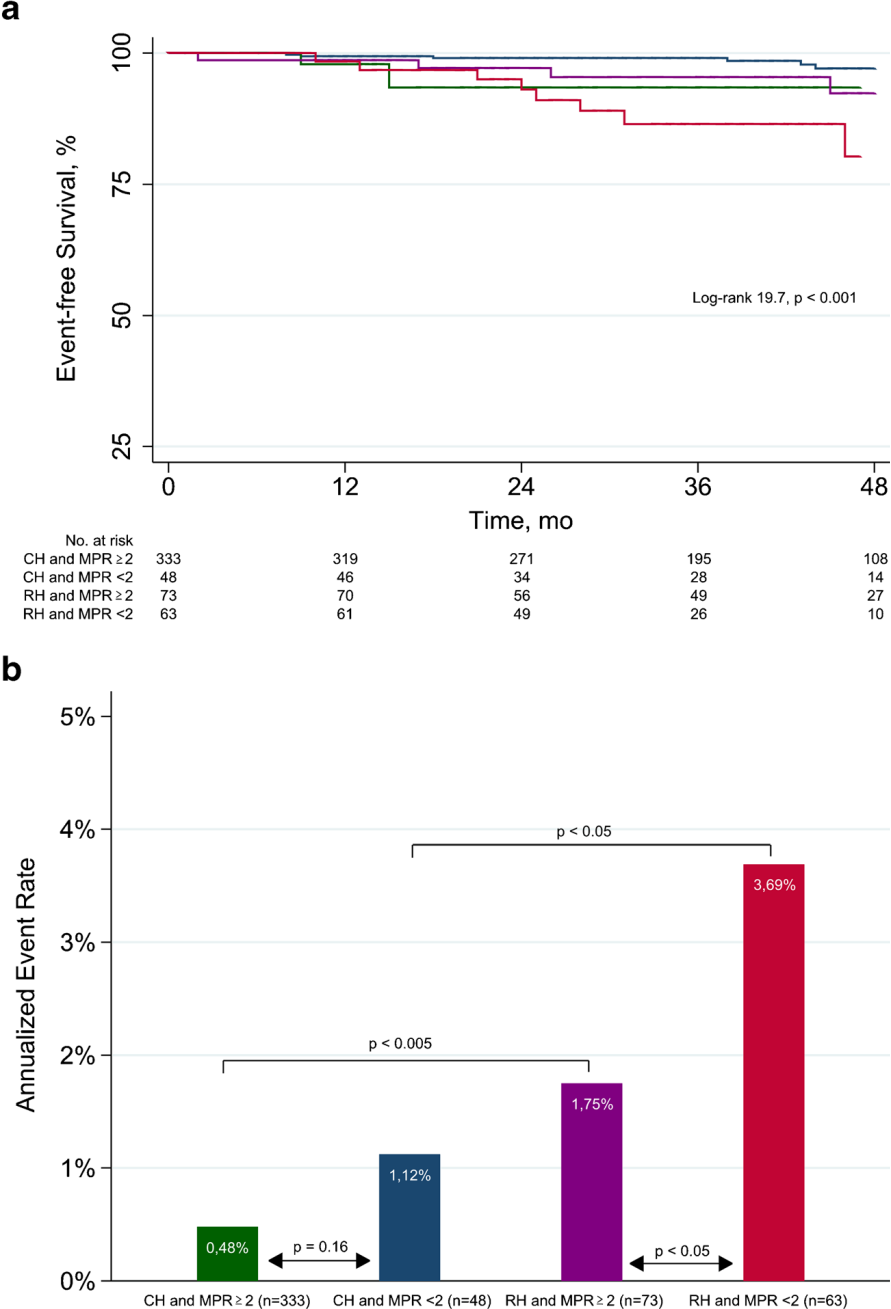
Event-free survival curves by Kaplan-Meyer analysis (**a**) and annualized event rates (**b**) according to hypertensive and coronary vascular function status. CH, controlled hypertension; RH, resistant hypertension; MPR, myocardial perfusion reserve

Univariable and multivariable Cox regression analyses are reported in Table 3. At univariable analysis, age, RH, LVEF reserve, CAC score categories, hyperemic MBF and MPR < 2 were significant predictors of events. At multivariable analysis, age, RH, and MPR < 2 were independent predictors of events. Event-free survival curves and AER according to hypertensive and MPR status are reported in Fig. 1. Patients with RH and reduced MPR had a lower event-free survival compared with those with CH and reduced MPR (*p* < 0.05). Interestingly, the event-free survival was similar in patients with RH and normal MPR and those with CH and reduced MPR (*p =* 0.47). The best outcome was observed in patients with CH and MPR ≥ 2. CH patients with reduced MPR showed no difference in AER as compared to those with normal MPR. Differently, RH patients with reduced MPR showed an AER significantly higher compared to those with normal MPR and compared to CH patients with reduced MPR (both *p* < 0.05).

**Table 3:** Univariable and multivariable analyses for prediction of cardiac events

	Univariable analysis		Multivariable analysis	
	Hazard ratio (95% CI)	*p* value	Hazard ratio (95% CI)	*p* value
Age	1.08 (1.04-1.13)	< 0.001	1.06 (1.02-1.11)	< 0.05
Male gender	0.86 (0.36-2.04)	0.75		
BMI	1.02 (0.96-1.09)	0.5		
Diabetes	1.77 (0.73-4.27)	0.21		
Hypercholesterolemia	1.18 (0.43-3.21)	0.75		
Smoking history	1.21 (0.49-3.01)	0.67		
Family history of CAD	1.05 (0.45-2.48)	0.91		
Symptoms	1.36 (0.55-3.37)	0.51		
LV hypertrophy	2.49 (0.81-7.61)	0.11		
Resistant hypertension	4.7 (1.95-11.33)	< 0.001	2.89 (1.06-7.57)	< 0.05
Baseline heart rate	0.99 (0.97-1.02)	0.48		
Peak stress heart rate	0.97 (0.93-1)	0.06		
Baseline SBP	1.01 (0.99-1.04)	0.14		
Peak stress SBP	1.01 (0.98-1.03)	0.48		
Baseline DBP	1 (0.97-1.03)	0.99		
Peak stress DBP	0.97 (0.93-1.01)	0.12		
LVEF reserve	0.88 (0.79-0.99)	< 0.05	0.91 (0.80-1.03)	0.14
CAC score categories	1.61 (1.15-2.26)	< 0.01	1.11 (0.80-1.67)	0.68
Baseline MBF	1.20 (0.24-6.16)	0.81		
Hyperemic MBF	0.49 (0.27-0.86)	< 0.05	0.97 (0.43-2.18)	0.95
MPR <2	3.63 (1.54-8.56)	< 0.005	2.58 (1.00-6.61)	< 0.05

*CI* confidence interval, *BMI* body mass index, *CAD* coronary artery disease, *LV* left ventricular, *SBP* systolic blood pressure, *DBP* diastolic blood pressure, *EF* ejection fraction, *CAC* coronary artery calcium, *MBF* myocardial blood flow, *MPR* myocardial perfusion reserve

### Change risk in time

**Fig. 2:**
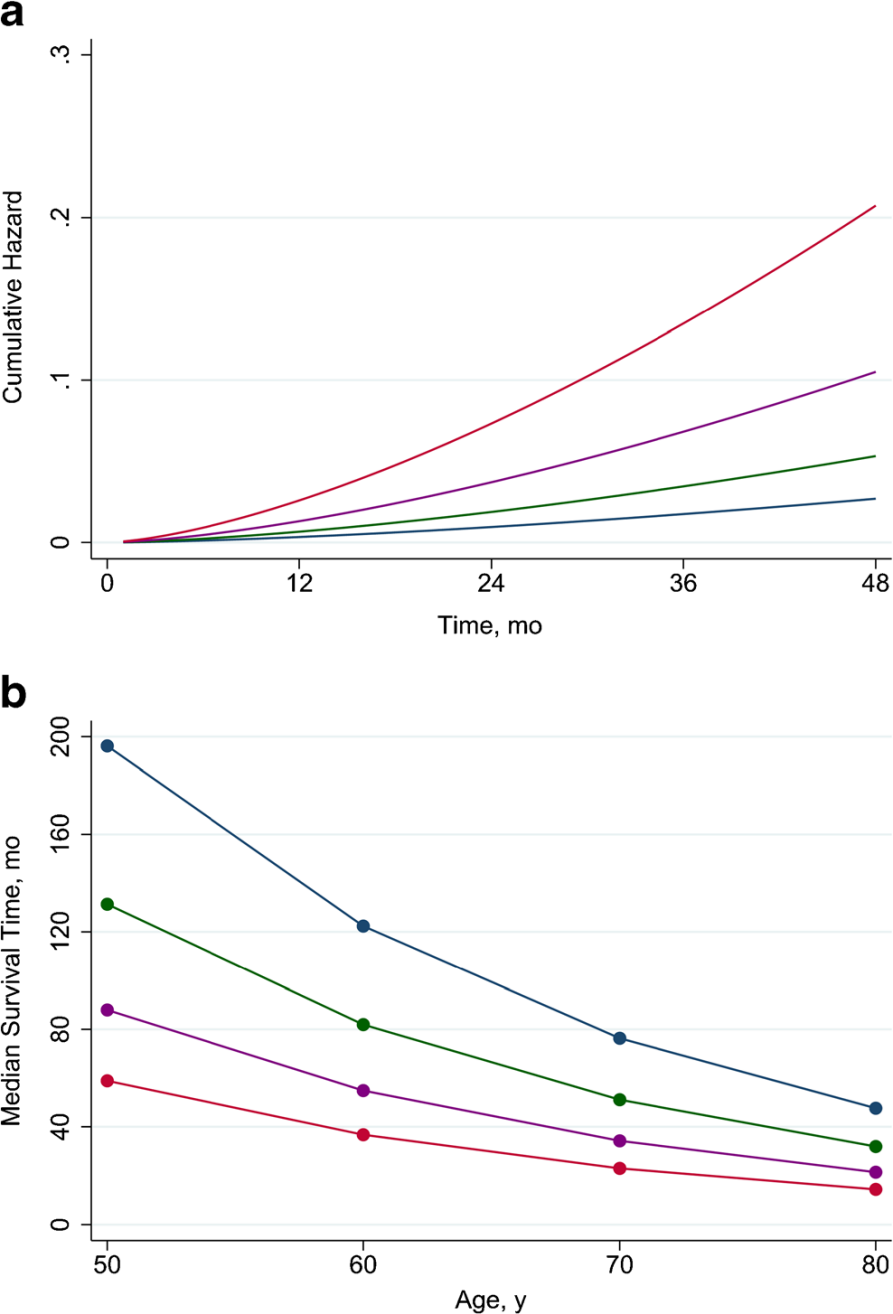
Predicted cumulative hazard by hypertension and coronary vascular function (**a**) and median survival time by age, hypertension, and coronary vascular function (**b**). Abbreviations as in Fig. 1

The cumulative hazard and the median survival time predicted by Weibull analysis are illustrated in Fig. 2. The survival model including hypertensive status and MPR as covariates revealed that the highest risk of cardiac events and the major risk acceleration were observed in patients with RH and reduced MPR. The probability of events was initially comparable in patients with RH and normal MPR and patients with CH and reduced MPR, but with a major risk acceleration for patients with RH over time. Patients with CH and normal MPR had the lowest probability of events. Yet, the predicted event-free survival time decreased with increasing age in all groups (Fig. 2b). For each age, the lowest value are detectable in RH patients with reduced MPR. However, the impact of reduced MPR decreases with advancing age.

## Discussion

This study first demonstrates that the presence of coronary vascular dysfunction, assessed by ^82^Rb PET/CT imaging, is associated with an increased risk of an adverse cardiac event in patients with RH [19]. Among cardiovascular risk factors, uncontrolled hypertension is one of the most critical, entailing an elevated risk of myocardial infarction, hemorrhagic or ischemic stroke, renal failure, and heart failure [19]. Regardless of other clinical and demographic characteristics, patients with RH have a higher risk of adverse cardiovascular events than patients with controlled hypertension; therefore, in these patients, any efforts should be performed toward improving outcomes [20]. Elevated BP is a well-established risk factor for cardiovascular events and lowering elevated BP has been demonstrated to reduce such risk [21, 22].

The duration and the grade of hypertension may influence the BP burden accumulated over time and this could explain the different incidence of complications in patients with and without RH [23]. However, published data advised that some factors other than BP burden may be quickening cardiovascular disease progression in patients with RH. In general, elevated BP causes vascular and cardiac structural and functional changes, such as the increased thickness of the carotid media and intima, cardiac hypertrophy, and ventricular diastolic dysfunction [24–26], which increase the risk of cardiovascular events.

It has been clearly reported that in absence of known CAD, patients with normal perfusion and RH have an evidently impaired microvascular function compared with controlled hypertension [9, 10] probably linked to the consequences of microvascular remodeling due to persistent high BP values. However, to our knowledge, this is the first study examining the prognostic value of coronary vascular dysfunction, assessed by ^82^Rb PET/CT, in patients with RH. Our results indicated that, despite a normal myocardial perfusion, patients with RH have a high risk of cardiovascular events. In particular, it also emerged that the inability to adequately increase MBF in response to stress identify RH patients with a substantially higher rate of events compared to those with normal coronary vasodilator reserve and remaining at higher risk also compared with CH patients with reduced MPR.

Previous studies demonstrated that the evaluation of coronary vasodilator function improves the prognostic assessment in different patients’ population [6–8]. Murthy et al. [6] demonstrated that among patients with moderate and severe renal impairment non-invasive assessment of vasodilator function provides incremental value in risk stratification, beyond clinical risk factors. Moreover, according to our findings, it has been reported that a reduced MPR is associated with a higher annual event rate over 3 years compared with normal MPR even in patients with normal perfusion [27]. Recently, Taqueti et al. [28] found that in symptomatic patients without flow-limiting epicardial CAD, impaired MPR is independently associated with diastolic dysfunction and adverse cardiovascular events, suggesting that coronary microvascular ischemia, in association with myocardial stiffness, may have an important role in the pathophysiology of the events.

Hybrid PET/CT, in addition to the evaluation of absolute MBF and MPR, is able to provide not only functional but also morphological information for the assessment of coronary status, such as quantification of the fat depot and CAC score measurement [29, 30].

Assante et al. [8] in a patient population with suspected CAD found that a high CAC score is associated with reduced MPR and that both CAC score ≥ 400 and MPR are significant predictors of cardiac events. When the two sides of the coin are evaluated to establish their prognostic value in patients with suspected CAD, it was observed that although both the extent of CAC and the presence of coronary vascular dysfunction are associated with an adverse cardiac event, after adjustment for clinical risk, only MPR improve risk assessment [31]. Furthermore, for any level of CAC score, the presence of reduced MPR is associated with adverse cardiovascular events. In the present study performed in patients with RH without any evidence of CAD according to normal perfusion imaging findings, CAC score even if resulted in a predictor of event at follow-up did not show an independent association with outcome in a multivariable model including clinical, hemodynamic, and imaging data. These results are in agreement with previous data demonstrating that CAC score is expression of macrocalcifications present in a more advanced stage of atherosclerosis providing information only on epicardial status. Whereas MPR reflects the integrity of both epicardial and microvascular circulation and is a marker of coronary dysfunction, the earliest step of atherosclerotic progression [31]. Our results strengthen the concept that the assessment of coronary vasodilator function, reflecting disease activity, is a more powerful indicator of cardiac risk than the total burden of calcified atherosclerosis. Between clinical variables, age resulted an independent factor in determining cardiovascular events. Age is already a recognized confounder as independent predictor of events in multivariable prediction models including all the traditional cardiovascular risk factors. However, in our multivariate model MPR remains an independent predictor of events despite such strong parameters as RH and age.The survival model including, hypertension status, MPR, and age as covariates showed that both CH and RH patients had a decrease in the survival time according to both age and MPR. The worst outcome was demonstrated in all the age categories in RH patients compared to CH patients and in the presence of reduced MPR. Moreover, the highest risk of cardiac events and the major risk acceleration were observed in patients with RH and reduced MPR. These results indicate that MPR assessment may identify an earlier stage of coronary dysfunction in the evolution of the atherosclerosis process. In fact, despite RH is a strong predictor of events, impaired MPR as assessed by perfusion PET even adds to the discrimination of higher versus lower risk patients. Thus, the functional evaluation of MPR in patients with RH could be a powerful marker of adverse cardiac events, reflecting pathophysiological changes, that could be corrected with an appropriate medical intervention. It has been suggested that hypertension can determine microvascular abnormalities and, as well, microvascular abnormalities can determine hypertension. Moreover, a kind of vicious circle may be determined in which microvascular damage caused by hypertension could help in sustaining or even exacerbating the rise in BP [32].

Our study could have clinical implications. First, considering that it remains unclear which are the processes involved in the higher risk of events of patients with RH compared to those with CH, evaluation of MPR could be useful to evaluate the effects of BP on coronary function. Second, a non-invasive measurement of coronary vascular function in patients with RH may help in identifying subjects with a higher cardiac risk acceleration over time, for whom a different therapeutic approach would be hypothesized. This study also has some limitations. In particular, despite the study population selected did not present a history of CAD and showed absence of myocardial perfusion abnormalities, angiographic data were not available. Thus, a possible role of coronary stenosis on reduced MPR values cannot be excluded.

## Conclusions

The highest risk of cardiovascular events is observed in subjects with RH and coronary vascular dysfunction. The findings suggest that the assessment of coronary vascular function may enhance risk stratification in patients with hypertension.

## Data Availability

Data will be made available from the corresponding author on reasonable request.

## Declarations

### Informed approval

Informed consent was obtained from all individual participants included in the study.

## References

[1] Williams B, Mancia G, Spiering W, Agabiti Rosei E, Azizi M, Burnier M, ESC Scientific sDocument Group (2018). 2018 ESC/ESH Guidelines for the management of arterial hypertension. Eur Heart J.

[2] Carey RM, Calhoun D, Bakris G, Brook RD, Daugherty SL, Dennison-Himmelfarb CR, American Heart Association Professional/Public Education and Publications Committee of the Council on Hypertension; Council on Cardiovascular and Stroke Nursing; Council on Clinical Cardiology; Council on Genomic and Precision Medicine; Council on Peripheral Vascular Disease; Council on Quality of Care and Outcomes Research; and Stroke Council (2018). Resistant hypertension: detection, evaluation, and management: a scientific statement from the American Heart Association. Hypertension.

[3] Prior JO, Allenbach G, Valenta I, Kosinski M, Burger C, Verdun FR (2012). Quantification of myocardial blood flow with 82Rb positron emission tomography: clinical validation with 15O-water. Eur J Nucl Med Mol Imaging.

[4] Zampella E, Acampa W, Assante R, Nappi C, Gaudieri V, Mainolfi CG (2018). Combined evaluation of regional coronary artery calcium and myocardial perfusion by (82)Rb PET/CT in the identification of obstructive coronary artery disease. Eur J Nucl Med Mol Imaging.

[5] Schindler TH, Schelbert HR, Quercioli A, Dilsizian V (2010). Cardiac PET imaging for the detection and monitoring of coronary artery disease and microvascular health. JACC Cardiovasc Imaging.

[6] Murthy VL, Naya M, Foster CR, Hainer J, Gaber M, Dorbala S (2012). Coronary vascular dysfunction and prognosis in patients with chronic kidney disease. JACC Cardiovasc Imaging.

[7] Murthy VL, Naya M, Foster CR, Gaber M, Hainer J, Klein J (2012). Association between coronary vascular dysfunction and cardiac mortality in patients with and without diabetes mellitus. Circulation..

[8] Assante R, Acampa W, Zampella E, Arumugam P, Nappi C, Gaudieri V (2017). Prognostic value of atherosclerotic burden and coronary vascular function in patients with suspected coronary artery disease. Eur J Nucl Med Mol Imaging.

[9] Völz S, Svedlund S, Andersson B, Li-Ming G, Rundqvist B (2017). Coronary flow reserve in patients with resistant hypertension. Clin Res Cardiol.

[10] Gaudieri V, Acampa W, Rozza F, Nappi C, Zampella E, Assante R (2019). Coronary vascular function in patients with resistant hypertension and normal myocardial perfusion: a propensity score analysis. Eur Heart J Cardiovasc Imaging.

[11] Dorbala S, Hachamovitch R, Curillova Z, Thomas D, Vangala D, Kwong RY (2009). Incremental prognostic value of gated Rb-82 positron emission tomography myocardial perfusion imaging over clinical variables and rest LVEF. JACC Cardiovasc Imaging.

[12] Cerqueira MD, Weissman NJ, Dilsizian V, Jacobs AK, Kaul S, Laskey WK, American Heart Association Writing Group on Myocardial Segmentation and Registration for Cardiac Imaging (2002). Standardized myocardial segmentation and nomenclature for tomographic imaging of the heart. A statement for healthcare professionals from the Cardiac Imaging Committee of the Council on Clinical Cardiology of the American Heart Association. Circulation..

[13] Klein R, Renaud JM, Ziadi MC, Thorn SL, Adler A, Beanlands RS (2010). Intra- and inter-operator repeatability of myocardial blood flow and myocardial flow reserve measurements using rubidium-82 pet and a highly automated analysis program. J Nucl Cardiol.

[14] Camici PG, Crea F (2007). Coronary microvascular dysfunction. N Engl J Med.

[15] Agatston AS, Janowitz WR, Hildner FJ, Zusmer NR, Viamonte M, Detrano R (1990). Quantification of coronary artery calcium using ultrafast computed tomography. J Am Coll Cardiol.

[16] Thygesen K, Alpert JS, Jaffe AS, Chaitman BR, Bax JJ, Morrow DA, Executive Group on behalf of the Joint European Society of Cardiology (ESC)/American College of Cardiology (ACC)/American Heart Association (AHA)/World Heart Federation (WHF) Task Force for the Universal Definition of Myocardial Infarction (2018). Fourth universal definition of myocardial infarction (2018). J Am Coll Cardiol.

[17] Lawless JF (2002). Statistical models and methods for lifetime data.

[18] Harrell FE (1997). Predicting outcomes: applied survival analysis and logistic regression.

[19] Yusuf S, Hawken S, Ounpuu S, Dans T, Avezum A, Lanas F, INTERHEART Study Investigators (2004). Effect of potentially modifiable risk factors associated with myocardial infarction in 52 countries (the INTERHEART study): case-control study. Lancet..

[20] Daugherty SL, Powers JD, Magid DJ, Tavel HM, Masoudi FA, Margolis KL (2012). Incidence and prognosis of resistant hypertension in hypertensive patients. Circulation..

[21] Acampa W, Rozza F, Zampella E, Assante R, Mannarino T, Nappi C (2020). Warranty period of normal stress myocardial perfusion imaging in hypertensive patients: a parametric survival analysis. J Nucl Cardiol.

[22] Ettehad D, Emdin CA, Kiran A, Anderson SG, Callender T, Emberson J (2016). Blood pressure lowering for prevention of cardiovascular disease and death: a systematic review and metaanalysis. Lancet..

[23] Pimenta E, Calhoun DA (2012). Resistant hypertension: incidence, prevalence, and prognosis. Circulation..

[24] Lonati L, Cuspidi C, Sampieri L, Boselli L, Bocciolone M, Leonetti G (1993). Ultrasonographic evaluation of cardiac and vascular changes in young borderline hypertensives. Cardiology..

[25] Escudero E, De Lena S, Graff-Iversen S, Almiron M, Cingolani HE (1996). Left ventricular diastolic function in young men with high normal blood pressure. Can J Cardiol.

[26] Kimura Y, Tomiyama H, Nishikawa E, Watanabe G, Shiojima K, Nakayama T (1999). Characteristics of cardiovascular morphology and function in the high-normal subset of hypertension defined by JNC-VI recommendations. Hypertens Res.

[27] Herzog BA, Husmann L, Valenta I, Gaemperli O, Siegrist PT, Tay FM (2009). Long-term prognostic value of 13N-ammonia myocardial perfusion positron emission tomography added value of coronary flow reserve. J Am Coll Cardiol.

[28] Taqueti VR, Solomon SD, Shah AM, Desai AS, Groarke JD, Osborne MT (2018). Coronary microvascular dysfunction and future risk of heart failure with preserved ejection fraction. Eur Heart J.

[29] Nappi C, Ponsiglione A, Acampa W, Gaudieri V, Zampella E, Assante R (2019). Relationship between epicardial adipose tissue and coronary vascular function in patients with suspected coronary artery disease and normal myocardial perfusion imaging. Eur Heart J Cardiovasc Imaging.

[30] Assante R, Acampa W, Zampella E, Arumugam P, Nappi C, Gaudieri V (2017). Coronary atherosclerotic burden vs. coronary vascular function in diabetic and nondiabetic patients with normal myocardial perfusion: a propensity score analysis. Eur J Nucl Med Mol Imaging.

[31] Naya M, Murthy VL, Foster CR, Gaber M, Klein J, Hainer J (2013). Prognostic interplay of coronary artery calcification and underlying vascular dysfunction in patients with suspected coronary artery disease. J Am Coll Cardiol.

[32] Serné EH, de Jongh RT, Eringa EC, IJzerman RG, Stehouwer CD (2007). Microvascular dysfunction: a potential pathophysiological role in the metabolic syndrome. Hypertension..

